# Out-of-Hospital Emergencies in Children Under Palliative Home Care

**DOI:** 10.3389/fped.2021.734181

**Published:** 2021-12-22

**Authors:** Holger Hauch, Naual El Mohaui, Johannes E. A. Wolff, Vera Vaillant, Sabine Brill, Emmanuel Schneck, Natascha Ströter, Ulf Sibelius, Peter Kriwy, Daniel Berthold

**Affiliations:** ^1^Palliative Care Team for Children, University Children's Hospital Giessen, Giessen, Germany; ^2^Pediatric Oncology, Cleveland Clinic, Cleveland, OH, United States; ^3^Anesthesiology and Intensive Medicine, University Hospital Giessen, Giessen, Germany; ^4^Pediatric Oncology, University Children‘s Hospital Giessen, Giessen, Germany; ^5^Palliative Care Team for Adults, University Hospital Giessen, Giessen, Germany; ^6^Institute of Sociology, Chemnitz University of Technology, Chemnitz, Germany

**Keywords:** emergency medical service, palliative home care, pediatric emergencies, cardiopulmonary resuscitation, do-not-resuscitate order

## Abstract

**Introduction:** Specialized palliative home care (SPHC) enables children and adolescents with life-limiting illnesses and complex needs to receive care at home. In addition to controlling symptoms and stabilizing the psychosocial situation, crisis anticipation is a component of SPHC. Since the establishment of the reporting SPHC team, parents have called for additional help from emergency medical services (EMS) in emergency situations with unexpected frequency. Children with life limiting diseases could undergo invasive procedures and unhelpful treatments with uncertain consequences. The questions arose as to which factors led to the involvement of the EMS in a palliative situation, what therapy was performed and what outcome could be reached.

**Methods:** Records of the pediatric SPHC patients and EMS call-outs in these children of the reporting SPHC-team in the central region of Hesse, Germany (population: 1.1 million) were retrospectively analyzed from 01.11.2014 to 01.05.2021. The causes of the call-outs, the existence of an emergency agreement, the National Advisory Committee for Aeronautics (NACA) score, EMS therapy and outcome were examined. Patient data included age, palliative-justifying diagnosis, duration and intensity of care, place of death and median overall survival (MOS) and palliative SHPC treatment.

**Results:** In total, 172 patients were analyzed during the study period. There were 27 EMS calls for a total of 20 patients/families (= EMS group). Palliative illness or a complication was the most frequent cause of call-outs. The patients in the EMS group were significantly less likely to have a DNR order, required more home visits and telephone calls and were under SPHC care for longer. There was a significantly higher proportion of crisis interventions at home visits. The children in the EMS group died less often from the underlying disease. Of the remaining 152 patients (= non-EMS group), a significantly higher proportion had a European home country.

**Conclusions:** Despite the introduction of the SPHC, parents still call the EMS. Good cooperation and joint training should be sought to prepare all those involved for future call-outs.

## Highlights

- This article shows a representative sample of children with life-limiting conditions in palliative home care.
- Despite a 24/7 on-call crisis program, parents call the EMS for emergencies.
- There were significant differences between the EMS and non-EMS group, which should enhance advance care planning with families and collaboration between palliative care and the EMS.

## Introduction

Since the introduction of specialized home palliative care (SPHC) (1) in Germany, children and adolescents with life-limiting illnesses are increasingly being cared for at home (2). Approximately 30 SPHC teams for children and adolescents have been established, allowing them to cover most of Germany (3). The palliative conditions are varied and include children with neuropediatric diseases, malformations, cancer, asphyxia, trauma, metabolic disorders, and other pediatric subspecialties (4). The SPHC aims to control the distressing symptoms, to contribute to psychosocial relief for the entire family, and to achieve stabilization of the home situation. An important element is the counseling of the caregivers, including with regard to possible crisis events and emergency situations (5). Usually, the SPHC is prescribed if the illness of the patient is in an advanced stage. For the reporting team it was not expected that parents repeatedly called the EMS. A potential risk is that the EMS team is confronted with a child with a severe chronic disease in a critical situation. In these rare cases very meaningful but also time critical decisions has to be made without all relevant information about the medical history.

The Franco-German EMS system aims to bring the doctor to the patient (6). An historical term to summarize emergency care is the “rescue chain.” (7) This chain contains two major parts: (1) prehospital; and (2) hospital care. The different strategies of the United States and Germany were that Germany preferred to focus on prehospital care, whereas the United States improved the second step in the emergency departments of the hospital (8). In Germany ambulances are staffed by emergency medical technicians (EMTs). In the state of Hesse of the reporting SPHC team there is a statutory regulation that in 90% of all emergencies an ambulance has to arrive within 10 min at the emergency location (9). Emergency medical doctors (EMDs) were transported by helicopters or specialized ambulances to the scene (10). Both EMDs and EMTs may be called to children who are terminally ill and in complex medical situations. However, the SPHC team, who knows the patients best and is well-prepared, covers a greater geographic area and therefore typically needs comparatively more time to arrive at the scene than the EMS. In addition, the vehicles of SPHC teams are not fitted with sirens. Thus, it is expected that parents of these seriously ill children call the EMS instead of or—in addition to—the SPHC team in emergencies that are considered time-critical.

Emergencies involving children are relatively rare in emergency services (11). Even less likely are call-outs for chronically or life-limitingly ill pediatric patients. The general proportion of children and adolescents in emergency ambulance calls has been found by various studies to be between 6.3 and 10% (12–14). Approximately 25% of pediatric emergencies are due to trauma (15) (e.g., fall from a changing table, traffic accident), and the remaining cases are acute illnesses (e.g., breathing disorders, febrile convulsion). In Germany, emergency physicians are often the primary responders in pediatric emergencies (16).

Since the establishment of outpatient palliative care structures is likely to result in an increasing number of patients receiving palliative therapy in the home environment (17, 18), it is likely that there will also be an increasing number of emergency situations and EMS responses for these patients. Often, children who receive palliative care at home suffer from chronic and very rare diseases. This makes time-sensitive decisions particularly difficult, especially when professional advance planning is lacking.

## Questions

- Which EMS operations occurred in the patients cared for in the SPHC, and how frequent were they?
- What treatments were given, and what was the outcome?
- Which possible associated factors can be identified that triggered the emergency call?

## Methods

The medical records of all patients treated at the single center pediatric SPHC-team from 01.11.2014 to 01.05.2021 were analyzed. The entry criterion for the study was the presence of a life-limiting disease that was advanced and caused severe symptoms. In addition, the need for care had to be complex (e.g., = at least 1 x/week home visit by a palliative care specialist and doctor was required, high psychosocial contextual factors). Variables such as sex/age, home country, residency (population and population density), palliative-justifying diagnosis, ACT group (19), ECOC (20), treatment with opioids/sedative drugs, and last status (alive/deceased) with date were recorded for all patients. In addition, the place of death (home/hospital/hospice/nursing home), existence of an advance directive for emergencies and the distance of the patient's residence from the SPHC team were documented. The (presumed) cause of death (tumor progression or progressive palliative disease/respiratory exhaustion/seizures/sepsis/unclear) was determined by two specialists on the basis of the course in the SPHC, the situation surrounding death and the findings of the post mortem examination.

A DNR order was considered in place if the parents did not want cardiopulmonary resuscitation, i.e., no chest compressions and no form of ventilation (invasive or non-invasive). The process of medical education and discussion with the parents and patients was structured accordingly the paper published by Rellensman et al. (21).The group of patients who were treated by an emergency physician/ambulance service (= EMS group) was compared with the group in which there was no EMS response (= non-EMS group). The care needs of both groups were also analyzed. For this purpose, the variables of duration of SPHC treatment, total number of home visits (HV) and telephone visits (TV) were recorded, in each case with or without the need for crisis intervention (CI). A CI was present if the patients were visited (HV-CI) or called (TV-CI) unscheduled in the home environment because of acute complaints. The measure of susceptibility to crises in care was calculated using the quotients (HV-CI/HV or TV-CI/TV). The quotients of numbers of HV (HV-CI) and TV (TV-CI)/duration of care in days were calculated to assess if a different need for contacts was associated with the period of care.

To check the representativeness of the study population, a comparison was made with anonymous data from all children and adolescents who were cared for in the SPHC system in the period from 2014 to 2018 across the entire federal state of Hesse (population: 6.26 million) (5).

The EMS call-outs were analyzed individually using the call-out logs. The following variables were collected: NACA score (22), year of the call-out and reported reason for the emergency call. From a medical point of view, the call-outs were causally classified as described by Wiese et al. (17):

1. Emergency independent of the palliative situation
2. Side effect of palliative therapy
3. Palliative-justifying underlying disease or its complications
4. Other reasons (e.g., family conflict)

It was necessary to consider these complex and rare situations to capture the parents' perspective. Here, during visits to the hospital or subsequent care at home, questions to the parents were asked—albeit not in a standardized way—about the motives or impulses after the emergency call was made, and this was summarized into the following categories:

A) Analogous to the parents' wish, the call-out served primarily to prolong life.
B) Symptom control was requested.
C) There was an emotional overload in the home environment.

Several responses from parents were also noted here. EMS therapy (e.g., need for cardiopulmonary resuscitation (CPR)), symptom control by medication) and outcome (e.g., transport to hospital, outpatient therapy, survival, continuing care) were recorded.

The study was approved by the Ethics Committee of the Justus Liebig University Giessen (file number: 88/2016). This study was verified with the German Registry of Clinical Studies (DRKS-ID: 00013318) and forwarded to the WHO Clinical Trials Registry.

The collected data of the patients and study participants were entered anonymously into databases of SPSS 25.0 software (IBM Inc. NY, USA) and analyzed. The parents/guardians gave consent for the study. All data was first analyzed descriptively. First, a descriptive analysis was performed to show differences in means and frequencies. To test differences between the EMS and non-EMS group *t*-tests were used for metric variables and Chi-square tests for categorical data. Significant differences are shown along the 5% level (two-sided). This is followed by the results of a multivariate analysis. All variables were processed appropriately for multivariate analysis. ACT groups 2 and 3 had to be combined because there was no variance on the dependent variable within category two. Furthermore, metric independent variables were tested for linearity. Because of the inverse u-shaped relationship between distance and EMS response, an additional squared term of distance in km was generated. Since the distribution of km is right skewed, an additional logarithmic term is used for regression analysis. The robustness of the model in different ranges of probabilities of the outcome was tested using the Hosmer-Lemeshow Test. This result is not significant and thus indicates good model fit.

## Results

### Characteristics of Patients

**Figure 1:**
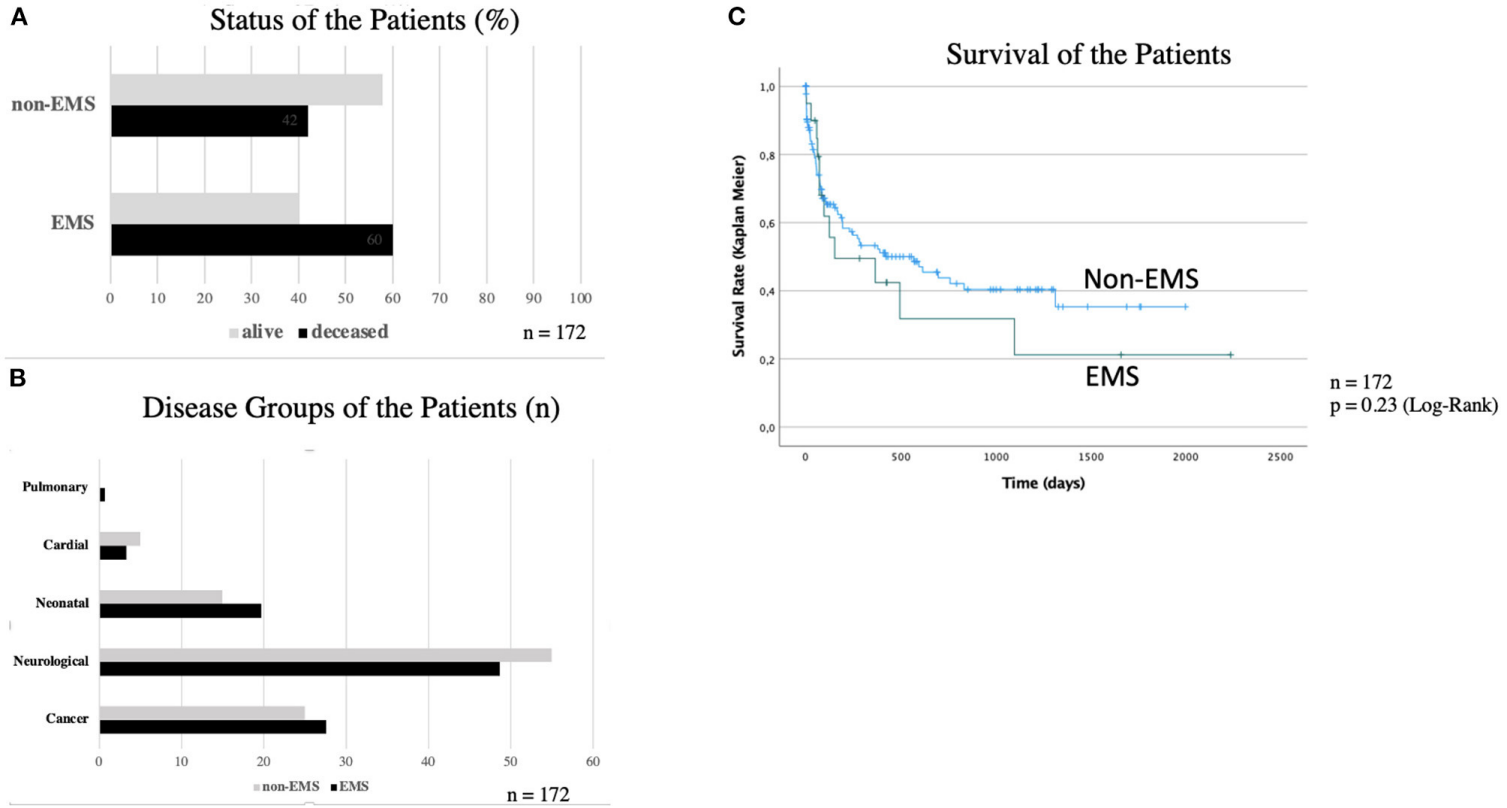
**(A–C)** Outcome and characteristics of the patients. **(A)** There was no difference between the patients' status (alive/deceased) between the EMS and non-EMS group. **(B)** Most of the patients suffered from neurological diseases and cancer. **(C)** Patients of the EMS group had a lower but not significant different survival.

During the 6-year and 6-month periods studied, a total of 173 patients were treated, and excluding one for an absence of consent, 172 were included in the study (Table 1). Of these patients, 20 (11.6%) had at least one EMS operation (EMS group). The 152 patients in the non-EMS group did not differ in terms of sex distribution, age, ACT groups, status, or place of death compared to the EMS group. There was also no difference from the larger cohort of all SPHC patients treated in the federal state of Hesse (6.3 million inhabitants) (5). Most of the patients suffered from neurologic diseases (48.8%), cancer (27.9%), congenital malformations (18.0%), cardiac diseases (2.9%), and prematurity (0.6%) (Figure 1B). The median ECOC status (EMS: 3; non-EMS: 3) was identical. The non-EMS group included significantly more patients whose home country was in Europe (non-EMS: 86.2%; EMS: 50%; *p* < 0.001). Additionally in the multivariate analysis could be shown that emergency services are 12% more likely to be called for children with an immigrant background than for children without an immigrant background (coeff. 1.97).

**Table 1:**
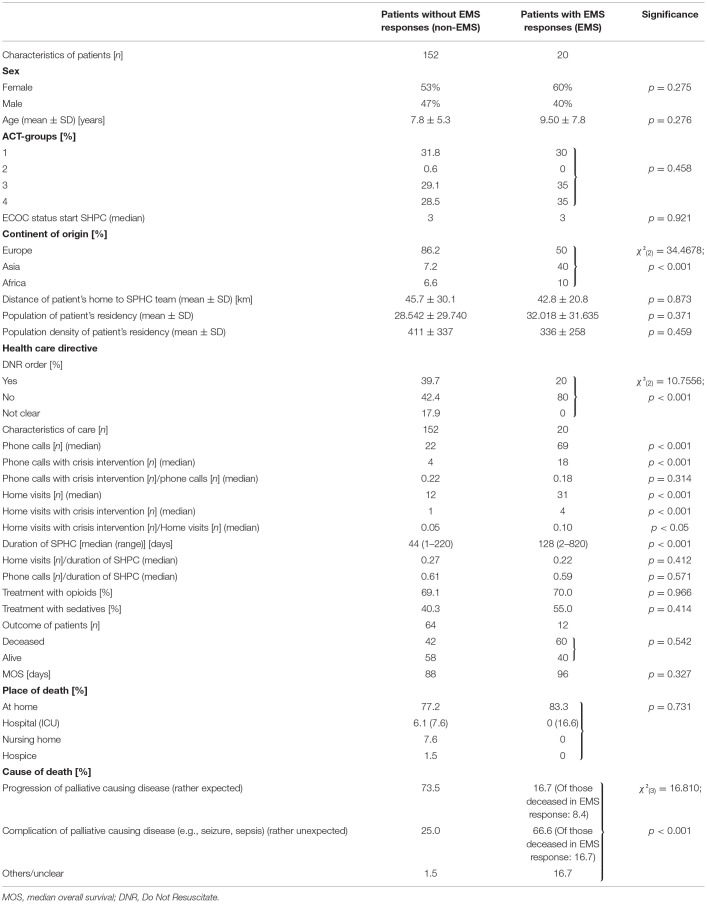
SPHC patients with and without EMS responses 2015–2020.

*MOS, median overall survival; DNR, Do Not Resuscitate*.

**Figure 2:**
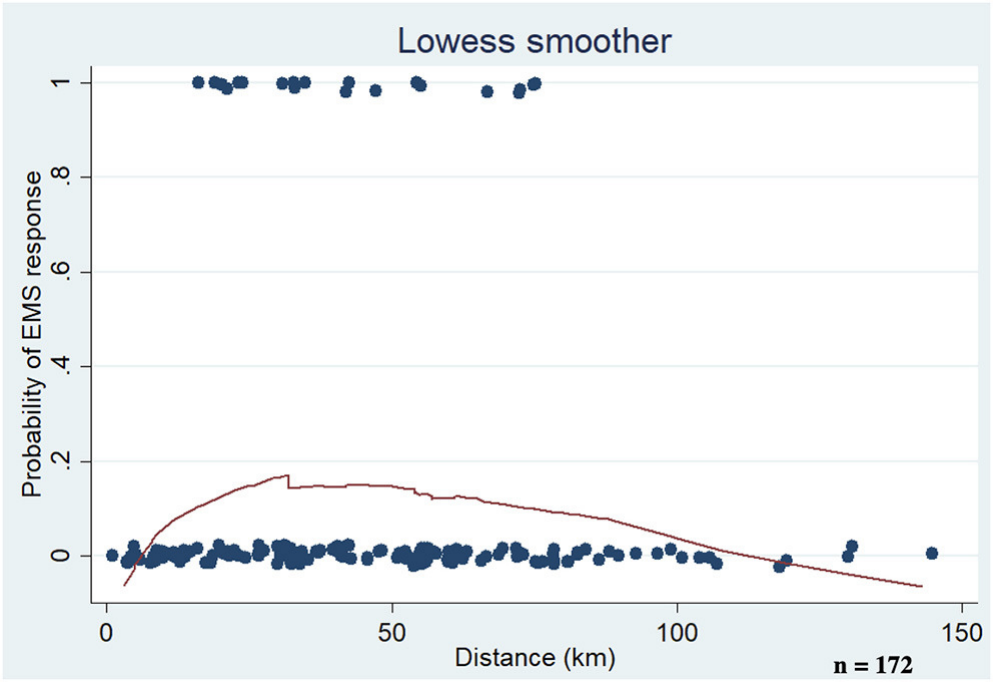
Influence of distance of the SHPC team to the patients' residency. In the multivariate calculation there is an inverted u-shaped relationship between distance in km and the use of emergency medical services. Up to a value of 32 km, the probability of calling an emergency medical service increases. However, this probability decreases continuously for distances >32 km. = single patient.

In the bivariate analysis the mean distance of the patients to the SPHC team did not differ between the two groups (non-EMS: 45.7 km ± 30.1; EMS: 42.8 km ± 20.8). But in the multivariate calculation there is an inverted u-shaped relationship between distance in km and the use of the EMS. The visualization of this functionality can be found in Figure 2 and the corresponding significance test is shown in Table 2. Up to a value of 32 km, the probability of calling the EMS increases. However, this probability decreases continuously for distances >32 km.

**Table 2:** Logistic regression (dependent variable 1 = EMS one or more times, 0 = no EMS).

	**Coeff**.	***T***
Female (a)	0.15	0.21
Age (in years)	0.07	1.66
ACT1 (b)	1.73	1.81
ACT4 (b)	1.61	1.79
DNR order	−1.81	−2.12^*^
Migration background	1.97	2.63^**^
Km (ln)	25.82	2.48^*^
Km squared (ln)	−3.61	−2.51^*^
Cause of death progression palliative causing disease (d)	0.12	0.13
Cause of death complication (d)	1.68	2.04^*^
Population density (low) (e)	1.02	1.25
Days in care (ln)	0.89	3.17^**^
Constant	−54.20	−2.76^*^
*p*	0.00	
Pseudo R-squared	0.43	
Hosmer-Lemeshow Test *p*	0.96	
Positive predictive value (dv = 1)	66.67	
Negative predictive value (dv = 0)	93.55	
*N*	172	

*This figure shows the results of the multivariate logistic regression. Regression coefficients are reported*.

* *p < 0.05;*

** *p < 0.01, (a) Ref.: male, (b) Ref.: ACT 2 & ACT 3, (c) Ref.: care level 1-3, (d) Ref.: still alive, (e) Ref.: middle or high population density (more than 150 people per square kilometer), source: own data, own calculation*.

The mean population of the residency (EMS: 32,018 ± 31,635; non-EMS: 28,542 ± 29,740) and the mean density of population (EMS: 336 ± 258; non-EMS: 411 ± 337) had no significant difference between both groups. In contrast, the effect of the DNR order is significant. Differences between the two groups were seen in the presence of a Do-Not-Resuscitate (DNR) order. In the non-EMS group, a DNR order was present in 39.7% of the cases (EMS 20%; *p* < 0.001). In the multivariate analysis could be shown the DNR reduced the likelihood of calling an emergency physician by 12% (coeff. −1.81). In addition, in the multivariate two control only variables are used: population density and the number of days children had spent in palliative care to date, or had spent until they died. Population density is not significant, and as the number of days in care rises, the likelihood of emergency services being called also increases significantly.

### Characteristics of Care

There were differences in both groups with regard to the duration and intensity of treatment. In the EMS group, the median treatment duration was 128 days (non-EMS: 44 days; *p* < 0.001). In the EMS group, more contacts were recorded with home visits (HV) (EMS: 31; NON-EMS: 12; *p* < 0.001) and telephone visits (TV) (EMS: 69; non-EMS: 22; *p* < 0.001). The median number of HV with crisis intervention characteristics (HV-CI) was also significantly more frequent in the EMS group (EMS: 4; non-EMS: 1; *p* < 0.001). The picture was similar for the TVs (TV-CI: EMS: 18; non-EMS: 4; *p* < 0.001). The EMS group showed a higher median proportion of crisis interventions/home visits (HV-CI/HV non-EMS ratio: 0.10; EMS: 0.05; *p* < 0.05). In the beginning of the home care the ECOC showed no difference (EMS: 3; non-EMS: 3). The usage of opioids (EMS: 70%; non-EMS: 69.1%; *p* = 0.966) and sedative drugs were very similar (EMS: 55%; non-EMS: 40.3%; *p* = 0.414).

### Outcome of the Patients

The patients status alive/deceased was not different in both groups (Figure 1A). The median overall survival (MOS) was not different between the two groups (EMS: 96 days; non-EMS: 88 days). The survival analysis (Kaplan Meier) showed no significant difference for both groups (Figure 1C). When considering the causes of death, the EMS group showed less frequent the progression of the underlying disease (EMS: 16.7%; non-EMS: 73.5%; *p* < 0.001) and more often an unclear death situation (EMS: 16.7%; non-EMS: 1.5%; *p* < 0.001). The multivariate calculation also controlled for the death of the children. Therefore, death is divided into two variants: (a) death of the basic disease and (b) death with complications of the underlying disease. When complications with the basic disease occur, the probability of calling emergency services is 11% higher (coeff. 1,68).

### Analysis of EMS Operations

**Figure 3:**
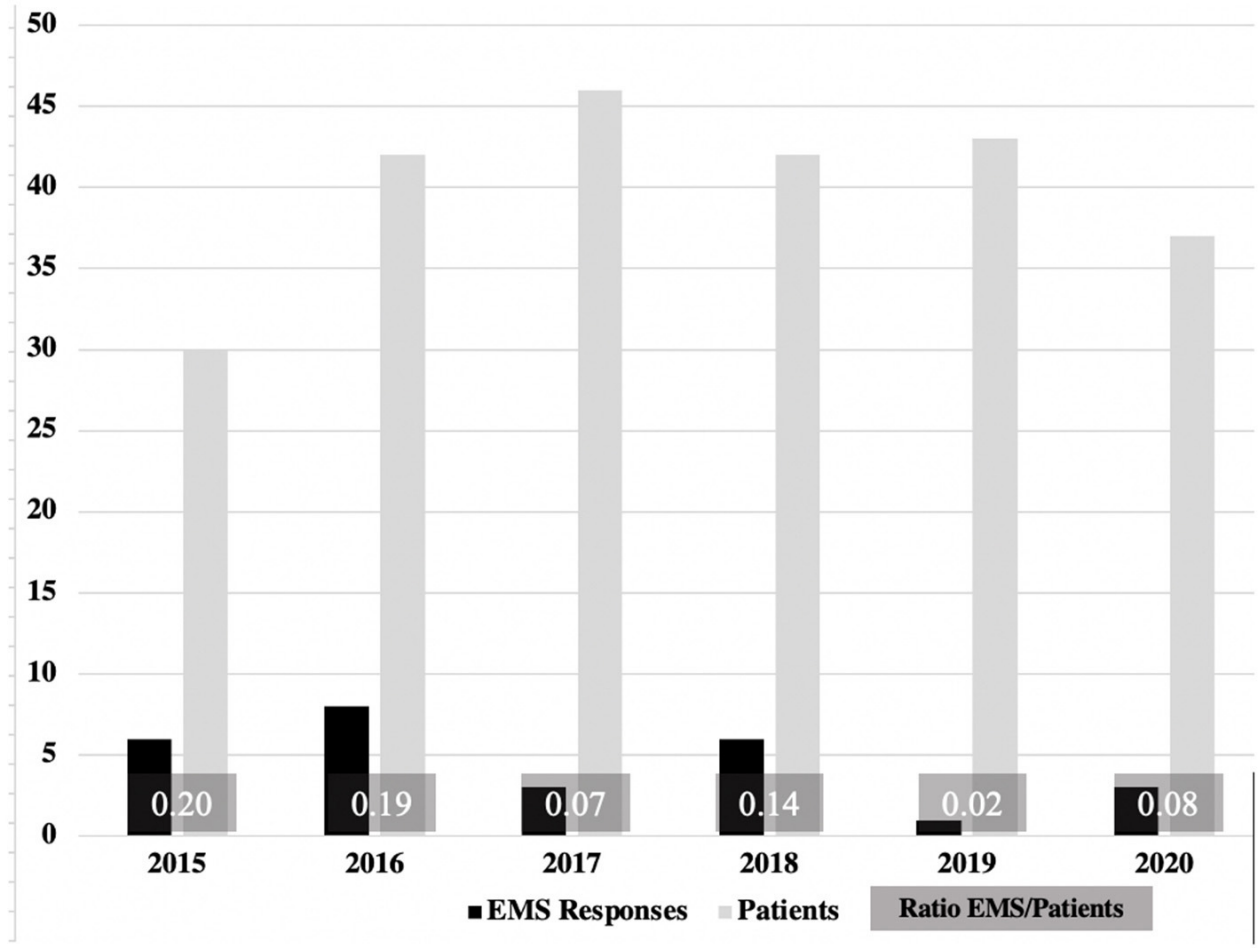
EMS responses in relation to the absolute patient numbers 2015–2020. There was a low but stable frequency of EMS operations. No reduction or significant changes were detectable in the years 2015–2020.

The 20 patients in the EMS group had a total of 27 emergency call-outs (Table 3). Two families called the emergency services three times, and three families called them twice. The most common reason for the call-out was the underlying disease or a related complication (20/27). A potential side effect of palliative therapy occurred once as the reason for the call-out was a grade 2 anaphylactic reaction to midazolam, which was successfully treated by epinephrine and steroids. Psychosocial crises led to emergency calls in 5/27 and independent emergencies in 2/27. All NACA classes were present. NACA Class 3 was the most frequently allocated at 11/27. A total of 19/27 emergency calls resulted in admission to the hospital. Four CPRs were documented (Table 3). In one case, an acute bolus event occurred, which was quickly recognized in a disability workshop, and CPR was initiated quickly. Neurologically, this patient showed no additional damage after successful CPR and died in her home environment with a stable quality of life 19 months later. Three children did not achieve a return of spontaneous circulation (ROSC). Two other children died in the intensive care unit after admission to the hospital with acute respiratory distress syndrome (ARDS). In 5 EMS responses, symptom control was performed (pain crises, seizures), and further care was provided by the SPHC team at a frequency of 21/27. From the parents' perspective, a total of 45 reasons for calling the emergency services were described for the 27 calls. The goal of prolonging life was mentioned by 16/45, the desire for symptom control by 8/45 and feeling emotionally overwhelmed by 21/45. Temporal observation over 6 years showed that there were persistent and regular emergency calls of the ambulance service for pediatric SPHC patients (Figure 3).

**Table 3:** All EMS responses of patients with SPHC treatment.

**EMS number**	**Patient number**	**Sex (F/M)/age at first SPHC admission (years)**	**Palliative diagnosis/ACT-group/existence of advance healthcare directive (Y/N, which)?**	**Emergency situation/(Groups)/NACA score**	**EMS treatment**	**Admission to hospital (Y/N)**	**Follow-up/Outcome**
1	1	F/22	Low Grade Glioma/ACT-1/(Y, CPR incl. ACLS)	Fall out of bed/(1; A)/III	i.v. analgesia, transport to hospital	Y	Discharge from hospital after 2 days, admission to SPHC treatment
2	1	F/22	Low Grade Glioma/ACT-1/(Y, CPR, incl. ACLS)	Sudden cardiac arrest, pulseless electrical activity/(3; A)/VII	CPR, ACLS	N	No ROSC, deceased at home, 16 days after first EMS response, suspected pulmonary embolism
3	2	F/13, Twin 1	Neuronal Ceroid-Lipofuscinosis type 2/ACT-3/(Y, CPR, incl. APLS)	Seizure (status epilepticus)/(3; B, C)/II	Oxygen supplementation, midazolam buccal, waited and transferred to SPHC team	N	Transfer to SPHC, deceased at home 30 days after EMS response
4	3	F/13, Twin 2	Neuronal Ceroid-Lipofuscinosis type 2/ACT-3/(Y, CPR, incl. APLS)	Suspected pneumonia/(3; B, C)/II	Oxygen supplementation, waited and transferred to SPHC team	N	Transfer to SPHC, deceased at home 42 days after EMS response
5	4	F/5	Krabbe's disease/ACT-3/ (Y, CPR, BLS, no intubation)	Pneumonia/(3; A)/III	Oxygen supplementation, transport to hospital	Y	Discharge from hospital after 5 days, follow-up SPHC treatment. Patient alive, 10-y old
6	5	F/28	Mitochondrial disease/ACT-3/(Y, CPR, incl. ACLS)	Sudden cardiac arrest, foreign body aspiration, pulseless electrical activity/(3; A)/VI	CPR, ACLS, ROSC, Transport to hospital	Y	Discharge from hospital after 14 days without further neurological impairment, follow-up SPHC treatment, deceased at home 19 months after EMS response
7	6	F/1	Low Grade Glioma/ACT-1/(Y, CPR, incl. APLS)	Suspected seizure, not confirmed/(4; A, C)/III	Transport to hospital	Y	Discharge from hospital after 2 days, follow-up SPHC treatment,
8	6	F/1	Low Grade Glioma/ACT-1/(Y, CPR, incl. APLS)	Suspected bacterial infection, not confirmed/(4, A, C)/III	Transport to hospital	Y	Discharge from hospital after 3 days, follow-up SPHC treatment
9	6	F/1	Low Grade Glioma/ACT-1/(Y, CPR, incl. APLS)	Suspected bacterial infection, not confirmed/(4, A, C)/III	Transport to hospital	Y	Discharge from hospital after 2 days, follow-up SPHC treatment. Lost to follow-up due to moving
10	7	M/16	High Grade Glioma/ACT-1/(Y, DNR order)	Seizure/(3, B)/IV	Intravenous application of midazolam, waited and transferred to SPHC team	N	Deceased at home 23 days after EMS response
11	8	F/14	Mucopolysaccharidosis type 3a/ACT-3/(Y, DNR order)	Seizure/(3, B, C)/IV	Intravenous application of midazolam, transport to hospital	Y	Discharge from hospital after 2 days, follow-up SPHC treatment
12	8	F/14	Mucopolysaccharidosis type 3a/ACT-3/(Y, DNR order)	Seizure/(3, B, C)/IV	Various intravenous anticonvulsive medications, transport to hospital	Y	Discharge from hospital after 4 days, follow-up SPHC treatment
13	8	F/14	Mucopolysaccharidosis type 3a/ACT-3/(Y, DNR order)	Seizure/(3, B, C)/III	Transport to hospital	Y	Discharge from hospital after 2 days, follow-up SPHC treatment, patient alive, 19-y old
14	9	M/17	Hypoxic ischemic encephalopathy (HIE)/ACT-4/(Y, CPR, incl. ACLS)	Family dispute/(4, C)/I	Transport to hospital	Y	Discharge from hospital after 2 days, follow-up SPHC treatment
15	9	M/17	Hypoxic ischemic encephalopathy/ACT-4/(Y, CPR, incl. ACLS)	Pneumonia/(3, A, C)/V	Oxygen supplementation, transport to hospital	Y	Admission to ICU, invasive ventilation, death in pulmonary failure 5 days after last EMS response
16	10	M/0.8	Hypoxic ischemic encephalopathy/ACT-4/(Y, full resuscitation, incl. APLS)	Suspected pneumonia/(3, A, C)/III	Oxygen supplementation, transport to hospital	Y	Discharge from hospital after 6 days, follow-up SPHC treatment
17	10	M/0.8	Hypoxic ischemic encephalopathy/ACT-4/(Y, full resuscitation, incl. APLS)	Bronchitis/(3, A, C)/III	Oxygen supplementation, transport to hospital	Y	Discharge from hospital after 4 days, follow-up SPHC treatment. Lost to follow-up due to stabilization
18	11	M/18	Leukemia/ACT-1/(Y, DNR order)	Dyspnea (3, B, C)/IV	Oxygen supplementation, waited and transferred to SPHC team	N	Transfer to SPHC, deceased at home 5 days after EMS response
19	12	F/1	Mitochondrial disease/ACT-3/(Y, full resuscitation, incl. ACLS)	Seizure (3, A, C)/IV	Oxygen supplementation, transport to hospital	Y	Admission to ICU, invasive ventilation, death in pulmonary failure, 14 days after last EMS response
20	13	F/6	Cardiomyopathy/ACT-1/(Y, full resuscitation, incl. APLS)	Dyspnea (3, C)/IV	Oxygen supplementation, waited and transferred to SPHC team	N	Transfer to SPHC, deceased at home 10 days after EMS response
21	14	F/4	Unknown syndrome/ACT-4/(Y, full resuscitation, incl. APLS)	Cardiac arrest (3, A, C)/VII	CPR, APLS, waited and transferred to SPHC team for necropsy and psychological crisis intervention	N	No ROSC, deceased at home
22	15	M/13	Charcot-Marie-Tooth/ACT-4/(Y, full resuscitation, incl. ACLS)	Family dispute/(4, C)/I	waited and transferred to SPHC team	N	Deceased at home 1 day after EMS response
23	16	M/8	Unknown syndrome/ACT-4/(Y, full resuscitation, incl. APLS)	Pneumonia/(3, A, C)/III	Oxygen supplementation, transport to hospital	Y	Discharge from hospital after 10 days, follow-up SPHC treatment, patient alive, 11-y old
24	17	M/9	Lennox-Gastaut Syndrome/ACT-4/(Y, DNR order)	Anaphylactic reaction (2, B)/IV	Intravenous application of a steroid, antihistamine drug and epinephrine	Y	Discharge from hospital, follow-up SPHC treatment, patient alive, 11-y old
25	18	F/1	Atypical Teratoid Rhabdoid Tumor/ACT-1/(Y, full resuscitation, incl. APLS)	Cardiac arrest (3, A, C)/VI	CPR, APLS, transport to hospital	Y	no ROSC, deceased at hospital
26	19	F/1	Hypoxic ischemic encephalopathy (HIE)/ACT-4/(Y, full resuscitation, incl. APLS)	Suspected pneumonia/(3, A, C)/III	Oxygen supplementation, transport to hospital	Y	Discharge from hospital after 4 days, follow-up SPHC treatment, patient alive, 2-y old
27	20	M/0.4	Diaphragmatic hernia and lung hypoplasia/ACT-1/(Y, full resuscitation, incl. APLS)	Suspected pneumonia/(3, A, C)/III	Oxygen supplementation, transport to hospital	Y	Discharge from hospital after 3 days. Lost from follow-up due to planned surgery

## Discussion

This study describes children with life-limiting diseases experienced emergency medical interventions even if a 24/7 on-call SPHC team is involved in their care. Prehospital emergencies with non-palliative children are sporadic and typically associated with conditions such as asthma, febrile seizures, or trauma (15, 23, 24). So, EMS operations with children in a palliative situation are even very rare. The problem is that the EMS teams are confronted with a complex situation, a rare disease and have to decide about e.g., mechanical ventilation within a short period of time. These decisions are difficult and the children have to bear the consequences. Thus, it was important to collect data to understand the mechanisms leading to a 911 call.

Data on the frequency of emergency call-outs in pediatric patients in the SPHC setting were limited to a pediatric oncology population. In one study, there were 4 EMS operations in the terminal phase in a total of 133 children (25).

In the study presented here, there were three EMS call-outs in pediatric oncology patients in the terminal phase (cases 2, 18, 25, Table 3). The majority of the other patients suffered from various diseases, including non-oncological diseases. Due to the similar characteristics of the large cohort of all children and those of adolescents treated in the SPHC in the federal state of Hesse (5) and the work of Hoell et al. (26), this study can be assumed to be representative of home-based palliative care in Germany.

Even though the proportion of EMS group of all patients was only 11.6%, there were regular emergency calls, most of which were caused by the palliative condition. Fortunately, there were no emergencies due to the potentially respiratory depressive effects of the drugs used (e.g., fentanyl, morphine, midazolam). However, in connection with a buccal administration of midazolam, a grade 2 anaphylactic reaction had to be treated after a seizure, which did not result in further injury or require intensive medical treatment. This side effect has been published repeatedly (27, 28) and is considered as a rare complication of midazolam (29). Our experience appears to be consistent with the limited data available in the literature. Even palliative sedation in the end-of-life care of children with cancer can be done safely in a domestic environment (30).

The hypothesis that with the increasing distance to the family the probability of EMS operations likewise increased was not confirmed. But we detected in the multivariate calculation an inverted u-shaped relationship between distance and the use of emergency medical service. To control a possible bias this result was controlled for population parameters but the u-shaped effect persisted. The collected data did not allow to identify a single cause for this result. It can only be speculated that families who resided in a greater distance to the university center possibly are used to the primary medical care.

A significant difference in the non-EMS group was the continent of the home country. Children whose home country was in Europe were significantly more often found in the non-EMS group. This effect was confirmed in the multivariate analysis. One explanation could be the existing language barrier to being able to describe a worsened condition to the SPHC team, e.g., on the phone. Other possible factors, such as cultural attitudes toward death and dying, religious aspects and a different basic biomedical understanding, could have an influence on parents calling emergency services (31). This issue cannot be addressed from the data collected. However, this result highlights the greater degree of vulnerability of families who cannot adequately express themselves in the language of the palliative team, for example. Since 2016, discussions about therapy goals have been held with families, mainly with a professional interpreter. Even then, the content to be conveyed and the proposed attitudes may not be adequately translated into the families' mother tongue. It will remain a challenge for SPHC teams to meet families with very different experiences, languages, and characteristics appropriately and with dignity to achieve the best possible outcome for sick patients.

One possible bias could have been a different readiness of the families to call the EMS because of the costs for an ambulance. In Germany, in 2020, 83.16 million residents were registered (32). 82.09 of them had a health insurance (73.36 of them had a statutory health insurance) (33). The costs for an EMS call are different because each county calculate the rates individually. But all the costs are paid by the insurance companies. Thus, there were no variations across the households relating to costs.

The attitude of parents not to forego life-prolonging measures was expressed in the EMS group by the lower rate of DNR orders. This result could be confirmed in the multivariate analysis.

In cases without a DNR order, the EMS team has sometimes seconds to decide whether to start CPR. In doubtful situations (unclear underlying disease, unclear duration of cardiovascular arrest, parents' wishes) it is well justifiable in the authors' view to start with initial life-saving measures, e.g., bag-mask ventilation. In cases of doubt, resuscitation should be started (34). The situation could be reassessed if more information was available (e.g., through an emergency agreement with the SPHC team).

On the other hand, resuscitation may not only imply having done everything for the deceased child to the end but also mean obstructing the inevitable dying in a home setting in the presence of the loving family and exposing the patient to potentially distressing treatment. This is not infrequently a dilemma for families who need much empathy and help. It is preferable for an emergency agreement to be integrated within the framework of an advance care planning concept (35).

Other authors described, in systematic reconsideration for DNR, that in 41% of the cases, a DNR was made in perioperative care of children with life-limiting conditions in a hospital setting (36). In our study, families with the intention to call the EMS had a DNR rate of 20%. The relevance of communication interventions to discuss code status with patients was recently shown in a meta-analysis (37). These interventions improve patient knowledge and may thus improve code status discussions and influence the patient's decisions regarding DNR status. This could prevent unnecessary EMS interventions.

The EMS group was also characterized by the fact that, in the event of the children's death, sudden or subacute events, such as respiratory exhaustion, were more frequent than the progression of a tumor or metabolic disease.

The EMS group had a higher level of need for care (longer duration of care, more contact with the team), partially due to a higher crisis potential (higher proportion of crisis interventions with home visits necessary). This could be explained that children in the EMS group might be more likely in unstable medical condition. Another mechanism of a greater proportion of CI in the home visits could be that there is more psychological burden in the families of the EMS group that resulted in an increased need or acute home visits in the night and even more EMS operations.

In the pediatric population, it would be useful for further prospective investigations to evaluate the factors (e.g., communication interventions, parents' understanding of the child's illness) leading to reliable advance care planning (ACP) and to give the families preparedness for an undesired emergency situation.

The primary gatekeeper, general practitioners or local pediatricians were not regularly involved in the home visits or in crisis intervention but should be more integrated.

The fact that parents called emergency services despite SPHC treatment could also simply be due to time pressure. In summary, it is an important realization for the authors that the resources of the EMS are very important in crisis situations, including because of their rapid availability. In a study by Wiese et al., it was shown that the involvement of an SPHC team in adults led to a lower rate of emergency calls to the EMS (38). This cannot be verified for the patients in this study because there was no control group, e.g., a sample of palliatively treated children without the support of an SPHC team.

In the future, the vulnerability of the families even with need of longtime care by a SPHC team, a language barrier, a certain distance to the palliative care team and a not defined therapy goal should be better taken into account in care planning to discuss the desired emergency care measures in detail with the caregivers. The EMS and the SPHC teams are not opponents and should seek and find the best solution together.

Cooperation at the sensitive interface between emergency services and SPHC teams is urgently needed for this highly vulnerable patient group. Ultimately, the authors are of the opinion that EMS and SPHC teams have the same goal, namely, to give patients the opportunity to live in a self-determined way and with dignity for as long as possible with a good quality of life. Future digital networks between the rescue coordination center and the palliative departments could help to improve the collaboration.

### Limitations of the Study

One limitation of the study is the small number of EMS operations. However, since these are very rare events, 27 fully documented and monitored responses in a representative cohort are not so small in relative terms. A systematic reappraisal is helpful to be better prepared for future call-outs.

In a retrospective study, 3.9% of the 1,583 analyzed call-outs with palliative patients were registered over 2 years (39). Children were involved in 0.2% of the call-outs (= 3 cases).

This study lacked an accurate perspective from the parents, who were not interviewed in a standardized way. The classification into causes of death was made according to findings from the individual courses of illness, the death situations and the post mortems carried out. However, an autopsy was not performed in any of the cases for reasons of reverence and lack of consequences.

A meaningful limitation is the retrospective design, which risks missing data. The authors hope to present the results of the prospective study “Eva in distress” soon; the study examines the process of the SPHC team and parents reaching an emergency agreement (40).

## Conclusions

Children with a life-limiting condition appear to play an underestimated role in EMS. Treatment of children with severe, chronic, and rare diseases is challenging, and specialized training should be offered to EMS professionals. Good collaboration on the interface between palliative and emergency medicine is needed to inform EMS about patients with the potential need for acute help in emergencies. Further investigations are required.

## Data Availability Statement

The raw data supporting the conclusions of this article will be made available by the authors, without undue reservation.

## Ethics Statement

The studies involving human participants were reviewed and approved by Ethics Committee, Fachbereich Medizin, Justus Liebig University, Giessen, Hesse, Germany. Written informed consent to participate in this study was provided by the participants' legal guardian/next of kin.

## Author Contributions

HH, JW, and DB conceived the study and designed the trial. HH and DB supervised the conduct of the trial and data collection. HH, NE, VV, and SB undertook recruitment of participating patients and managed the data and including quality control. HH, DB, and PK provided statistical advice on the study design and analyzed the data. HH drafted the manuscript and takes responsibility for the paper as a whole. VV, SB, US, NS, ES, and PK contributed substantially to its revision. All authors contributed to the article and approved the submitted version.

## Funding

This study was approved by the German Register for Clinical Trials (Study No. DRKS00013318).

## Conflict of Interest

The authors declare that the research was conducted in the absence of any commercial or financial relationships that could be construed as a potential conflict of interest.

## Publisher's Note

All claims expressed in this article are solely those of the authors and do not necessarily represent those of their affiliated organizations, or those of the publisher, the editors and the reviewers. Any product that may be evaluated in this article, or claim that may be made by its manufacturer, is not guaranteed or endorsed by the publisher.

## Abbreviations

AHCD: Advanced Health Care Directive
APLS: Advanced Pediatric Life Support
CPR: Cardio-pulmonary Resuscitation
DNR: Do-Not Resuscitate-Order
DRKS: German Registry of Clinical Studies
EMP: Emergency Medical Physician
EMS: Emergency Medical Service
EMT: Emergency Medical Technician
GP: General Practitioner
ICU: Intensive Care Unit
ROSC: Return of Spontaneous Circulation
SD: Standard Deviation
SPHC: Specialized Palliative Home Care.
